# Progression of Diabetic Retinopathy and Declining Renal Function in Patients with Type 2 Diabetes

**DOI:** 10.1155/2020/8784139

**Published:** 2020-07-26

**Authors:** AJin Cho, Hayne Cho Park, Young-Ki Lee, Young Joo Shin, So Hyun Bae, Hakyoung Kim

**Affiliations:** ^1^Department of Internal Medicine, Kangnam Sacred Heart Hospital, Seoul, Republic of Korea; ^2^Hallym University Kidney Research Institute, Seoul, Republic of Korea; ^3^Department of Ophthalmology, Kangnam Sacred Heart Hospital, Seoul, Republic of Korea

## Abstract

**Background:**

At a university hospital in Korea, we conducted a retrospective study to determine the association of the progression of diabetic retinopathy (DR) with declining renal function in type 2 diabetes.

**Methods:**

We included a total of 1527 patients with type 2 diabetes who followed up in our diabetes clinic and underwent fundus photographic examinations from August 2006 to February 2014. DR was assessed by retinal ophthalmologists using comprehensive ophthalmologic examinations.

**Results:**

The baseline prevalence of nonproliferative DR (NPDR) and proliferative DR (PDR) was 26.5% and 14.7%, respectively. Among 1303 patients with no DR and NPDR, 134 (10.3%) patients progressed to NPDR or PDR. The progression group had longer duration of diabetes, higher fasting plasma glucose, higher HbA1c, and a higher rate of ≥20% decline in eGFR during the follow-up period. After multivariate analysis, ≥20% decline in eGFR (odds ratio 2.553, 95% CI 1.219-5.348, *p* = 0.013) was an independent risk factor for progression of DR in patients with NPDR.

**Conclusion:**

Declining renal function was independently associated with DR progression in patients with NPDR, suggesting that investigation of DR status should be recommended for patients with declining renal function.

## 1. Introduction

Type 2 diabetes has become a pandemic disorder and its increase in prevalence raises concerns worldwide [1]. Diabetes mellitus (DM) causes microvascular complications (retinopathy, nephropathy, and neuropathy) that are major causes of morbidity and mortality [2]. Diabetic retinopathy (DR) is an important microvascular complication and is the most common cause of preventable blindness in adults [3]. Diabetic nephropathy is a leading cause of chronic kidney disease (CKD) and end-stage renal disease [4]. The retina and the kidney share similar microvascular complications resulting from DM [5, 6].

The association of microalbuminuria and DR has been reported in patients with type 1 diabetes. Among patients with type 1 diabetes who have nephropathy, more than 95% already have DR [7]. Several studies reported close associations between renal dysfunction and DR in patients with type 1 diabetes [8–10]. In type 2 diabetes, the association appears much weaker than in type 1 diabetes. The situation is more complicated for patients with type 2 diabetes because they are also susceptible to parenchymal renal diseases other than classic diabetic glomerulosclerosis [11]. In patients with type 2 diabetes, CKD occurs in the absence of DR or microalbuminuria [12].

Several studies have shown that DR severity was significantly associated with reduced kidney function and increased risk of CKD in type 2 diabetes [13–15]. Although some cross-sectional studies have reported associations between renal function and prevalent DR, there are scarce data about relationships between DR progression and renal dysfunction in patients with type 2 diabetes. In this study, we investigated whether progressive renal function decline affects DR progression in type 2 diabetes.

## 2. Patients and Methods

### 2.1. Study Population

We enrolled patients with type 2 diabetes from the diabetes clinic in the Department of Endocrinology of Kangnam Sacred Heart Hospital who underwent fundus photographic examinations for DR and whose renal profiles were studied between August 2006 and February 2014. We excluded patients with estimated glomerular filtration rate (eGFR) < 15 ml/min/1.73 m^2^ and without follow-up renal profiles and fundus exam obtained more than 3 months after the first evaluation. This retrospective, observational study was performed in accordance with the Declaration of Helsinki and approved by the Institutional Review Board of Hallym University Kangnam Sacred Heart Hospital (IRB No: 2018-01-030). We could not obtain the informed consent for the patients because we used deidentified and retrospective data. This issue also was confirmed by the hospital's Institutional Review Board.

### 2.2. Measurement

Baseline characteristics, including demographics (age, gender), medical history (hypertension, diabetes, duration of diabetes), and laboratory variables were collected at the time of first DR assessment. Weight and height were assessed and body mass index (BMI) was calculated. Blood pressure was measured with a sphygmomanometer after 5 min of rest. Hemoglobin A1c (HbA1c) was measured using a method that was NGSP certified and standardized to the DCCT assay. A standard urine dipstick was used to measure albuminuria qualitatively. Serum creatinine was measured using the modified Jaffe method. Based on the serum creatinine concentration, the eGFR was calculated using the four-variable equation from the Modification of Diet in Renal Disease study [16]. Patients then were assigned to one of the following eGFR categories: G1 (≥90), G2 (60–89), G3 (30–59), and G4 (15–29 ml/min/1.73 m2) [17]. We defined categorical variables for significant renal function decline, using ≥20% decline in eGFR during the follow-up period [18, 19].

### 2.3. Determination of Diabetic Retinopathy

DR presence was assessed by retinal ophthalmologists who had no knowledge of the clinical details using slit-lamp examination, indirect ophthalmoscopy, and/or fluorescein angiography. Patients were classified into the following categories: (1) normal: no apparent sign of DR; (2) non-proliferative DR (NPDR): including microaneurysms, hard exudates, intraretinal hemorrhages, venous beading, or prominent intraretinal microvascular abnormality; and (3) proliferative DR (PDR): including retinal or optic disk neovascularization, vitreous hemorrhage, or preretinal hemorrhage, according to the Global Diabetic Retinopathy Project Group [20]. The presence and severity of DR in a participant were determined based on the eye showing the worst retinopathy. DR progression was defined as a change either from no DR progress to NPDR or from NPDR to PDR.

### 2.4. Statistical Analysis

Data were expressed as mean (standard deviation) for continuous variables and as numbers of cases and percentages for categorical variables. Patients were stratified by DR grade and progression of DR status. Differences between the groups were assessed using the chi-squared test for dichotomous factors and one-way ANOVA for continuous factors. Logistic regression analyses with stepwise variable selection were performed to assess the independent association of progression of DR. Univariate logistic regression models were employed first, followed by multivariate logistic regression models with adjustment by covariates that were significant (*p* < 0.05) in the univariate analysis. All *p* values were two-sided, and *p* < 0.05 was considered statistically significant. Statistical analyses were performed using SPSS version 18.0 (SPSS Inc., Chicago, IL).

## 3. Results

### 3.1. Baseline Characteristics

Clinical characteristics of the patients according to the retinopathy group are shown in Table 1. Among the 1527 patients, the mean age was 58 ± 11 years, the mean duration of diabetes was 9.2 ± 7.7 years, and 47.4% were men. Their mean baseline eGFR was 76.3 ± 23 ml/min/1.73 m^2^ and mean HbA1c was 7.8 ± 1.8%. Baseline eGFR of 21% of the patients was 15–59 ml/min/1.73 m^2^. A total of 86% of patients had no albuminuria. Among all patients, 898 (58.8%) had no signs of DR, 405 (26.5%) had NPDR, and 224 (14.7%) had PDR. Patients with DR had a longer duration of diabetes (*p* < 0.001), lower BMI (*p* < 0.001), higher systolic blood pressure (*p* = 0.014), and higher levels of fasting plasma glucose (FPG) (*p* < 0.001) and HbA1c (*p* < 0.001).

### 3.2. Prevalent Diabetic Retinopathy and Renal Function

**Figure 1:**
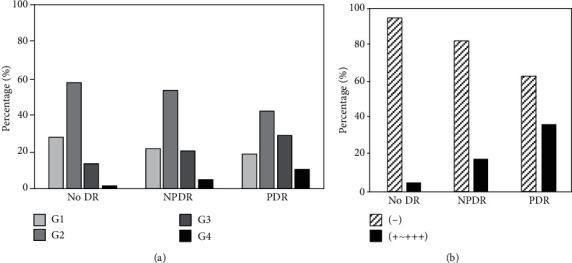
Prevalent diabetic retinopathy and renal function. (a) eGFR category: G1 (≥90), G2 (60–89), G3 (30–59), and G4 (15–29 ml/min/1.73m^2^) and (b) albuminuria. Abbreviation: eGFR: estimated glomerular filtration rate.

Figure 1 shows prevalence rates of eGFR categories by DR grades. Majority of patients were in G2: 57.7% of no DR, 53.3% of NPDR, and 42% of PDR. Patients with higher DR grade had a higher prevalence rate of G3 (13.4% of no DR, 20.5% of NPDR, and 29% of PDR, *p* < 0.001) and G4 (1.1% of no DR, 4.7% of NPDR, and 10.3% of PDR, *p* < 0.001) as well as a higher rate of albuminuria (5.3% of no DR, 17.8% of NPDR, and 36.8% of PDR, *p* < 0.001).

### 3.3. Progression of Diabetic Retinopathy and Associations with Declining Renal Function

The mean follow-up period was 4.0 ± 2.0 years. Among 1303 patients with no DR and NPDR, 134 (10.3%) progressed to NPDR or PDR. The characteristics of patients according to progression of DR are shown in Table 2. The progression group had longer duration of diabetes (8.5 ± 7.5 years vs. 10.6 ± 7.5 years, *p* = 0.002), higher FPG (145.2 ± 64.1 mg/dl vs. 173.6 ± 83.8 mg/dl, *p* < 0.001), and higher HbA1c (7.6 ± 1.7% vs. 8.7 ± 2.0%, *p* < 0.001). Baseline eGFRs were not significantly different between groups. Nevertheless, the progression group had a higher rate of baseline albuminuria (8.2% vs. 18.8%, *p* < 0.001).

**Figure 2:**
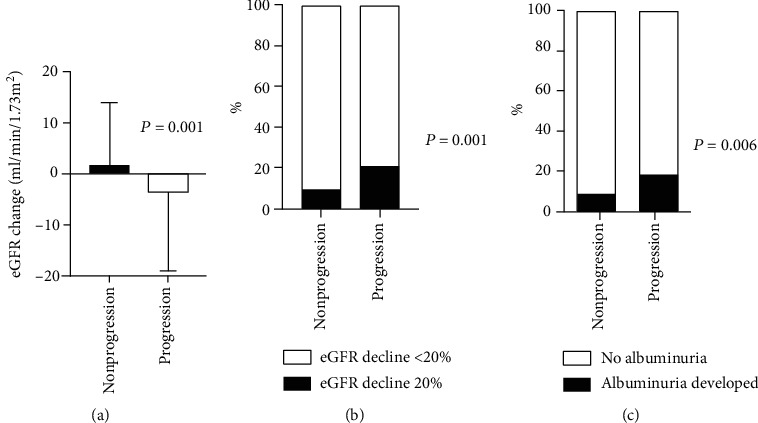
Changes of renal function in both groups. (a) Mean eGFR change. (b) Rate of ≥20% decline in eGFR during the follow-up period. (c) Albuminuria development. Abbreviation: eGFR: estimated glomerular filtration rate.

Figure 2 shows the changes of renal function in both groups. During the follow-up period, mean eGFR changes in the progression and nonprogression groups were –4.87 ± 24.72 and 1.98 ± 20.92, respectively (*p* < 0.001). The progression group had a higher rate of ≥20% decline in eGFR during the follow-up period (10.4% vs. 20.9%, *p* < 0.001). Among 1074 patients who had no proteinuria, 1036 had follow-up urine dipstick tests. A total of 99 out of 1036 (9.6%) developed albuminuria during follow-up. Sixteen patients of 91 (17.6%) in the progression group and 83 of 945 (8.8%) in the nonprogression group developed albuminuria (*p* = 0.006).

We performed binary logistic regression analysis to identify factors associated with progression of DR. On univariate analysis, longer duration of diabetes (odds ratio (OR) 1.034, 95% CI 1.012–1.057, *p* = 0.003), higher FPG (OR 1.005, 95% CI 1.003–1.007, *p* < 0.001), higher HbA1c (OR 1.318, 95% CI 1.210–1.435, *p* < 0.001), albuminuria (OR 2.576, 95% CI 1.543-4.301, *p* < 0.001), and ≥20% decline in eGFR (OR 2.288, 95% CI 1.449–3.613, *p* < 0.001) were associated with progression of DR. Next, we performed multivariate analysis for progression of DR according to baseline DR status (Table 3). Longer duration of diabetes (OR 1.062, 95% CI 1.025–1.100, *p* = 0.001), higher HbA1c (OR 1.353, 95% CI 1.191–1.537, *p* < 0.001), and albuminuria (OR 2.791, 95% CI 1.244-6.263, *p* = 0.013) were associated with progression of no DR to NPDR. In patients with NPDR, a ≥20% decline in eGFR decline (OR 2.553, 95% CI 1.219–5.348, *p* = 0.013) and younger age (OR 0.966, 95% CI 0.937–0.995, *p* = 0.023) were independent risk factors for progression to PDR.

## 4. Discussion

This study evaluated the association of progression of DR and declining renal function in patients with type 2 diabetes. We found that prevalent DR severity was associated with decreased eGFR and albuminuria. Furthermore, a ≥20% decline in eGFR was independently associated with the progression of NPDR to PDR. Duration of diabetes, albuminuria, and HbA1c were independent risk factors for progression of no DR to NPDR. The results of this study suggest that investigation of DR status should be recommended for patients with declining renal function, especially for NPDR patients. This result also supports the notion of a shared pathogenetic mechanism of DR and diabetic nephropathy in patients with type 2 diabetes.

Both the retina and the kidney are supplied by very small vessels. The anatomical similarities in the vascularization of the retina and the kidney give rise to complications of diabetes in the small vessels that appear in both organs. The microvascular changes in both organs are thought to be initiated by chronic hyperglycemia, followed by the progressive narrowing and eventual occlusion of vascular lumina, subsequently leading to inadequate perfusion of affected tissues [21, 22].

There are several studies about the association between DR and kidney function [13, 23]. A study of 523 participants with type 2 diabetes showed that increasing DR severity was significantly associated with reduced kidney function and increased risk of CKD [23]. These associations were independent, and it was suggested that the assessment of DR may provide useful information about renal function and the risk of kidney disease. However, few data have been reported on the association between changes in GFR and progression of DR, and most of these studies analyzed baseline GFR as the measurement of renal function. Tam et al. found that baseline eGFRs were not significantly different between the subjects who had development/progression of DR and those without [15]. In this study, the association between baseline eGFR and progression of DR was not significant. However, the progression of DR was associated with a decline in eGFR in patients with NPDR. Common mechanisms of retinal and renal vascular changes in diabetic patients support this result.

We showed that risk factors of DR development were different from ones of DR progression. The difference can be explained to originate from difference of their natural course and clinical characteristics. There are few studies to explore the DR natural course. In the previous studies, development from no DR to NPDR was estimated to take about 14 years, while DR progression rate was considerably fast, around 4 years, for patients who progressed to sever e form of DR [24, 25]. Schreur et al. and Tseng et al. also reported that risk factors associated with DR onset and progression in diabetes patients are different [26, 27]. In the studies, patients with baseline preexisting DR had older age, longer DM duration, higher FPG, and higher HbA1c level than ones with no baseline DR. This study demonstrated differences of baseline characteristics between no DR and NPDR groups, too.

Few cohort studies have investigated progression of DR in patients with type 2 diabetes, and most of these found that microalbuminuria, duration of diabetes, glycemic level, and baseline blood pressure were important independent predictors of DR incidence and progression [15, 28, 29]. The results of the present study are comparable with those of previous works. The duration of diabetes, albuminuria, and HbA1c were associated with the progression of DR from no DR to NPDR. Younger age was an independent risk factor for progression of NPDR to PDR, which is different from other previous studies [26, 30, 31]. In a population-based study, younger age at diagnosis was associated with increasing risk of incidence and progression of DR [32]. Another study reported that incidence rates of both developments of NPDR and progression from NPDR to PDR in early-onset DM was high, when compared with general diabetes population [33]. Clinical implication of the patients who were young and had a progressive form of DR needs further studies in the future.

This study had several limitations. First, there is the inherent weakness of all studies with a retrospective design, namely, the use of data from past medical records. Thus, we cannot propose causal associations or prediction of declining renal function in patients with DR. Future prospective cohort studies are needed to determine whether the progression of renal disease predicts progression of DR in patients with type 2 diabetes. Second, DR classification in this study was based on graders' discretion which might result in potential bias. However, each experienced retinal specialist determined DR grades according to globally accepted guideline. Third, we cannot know for certain whether risk factors are causing retinopathy progression or merely represent markers of disease progression. Fourth, because we were not able to collect dipstick results from all participants, we performed multivariate analysis for progression of DR excluding those without dipstick results. Furthermore, we could not obtain quantitative results of albuminuria such as urine microalbumin-to-creatinine ratio. Fifth, we did not collect CKD duration data and could not analyze its contribution as a prognostic factor.

In conclusion, we identified several factors associated with the progression of DR, including HbA1c level, duration of diabetes, younger age, and declining renal function. These factors were associated with DR progression in different ways, depending on baseline DR status. The decrease of renal function was associated with progression of DR, especially in patients with NPDR. This result supports the notion that an individualized screening schedule according to the individual patient's risk might be needed. Future prospective cohort studies are needed to evaluate the predictive value of renal disease for the development and progression of DR.

## Figures and Tables

**Table 1:** Baseline characteristics of type 2 diabetes according to retinopathy status.

Characteristics	All participants, *N* = 1527	No DR, *N* = 898	NPDR, *N* = 405	PDR, *N* = 224	*p* value
Age (years)	58 ± 11	58 ± 11	60 ± 11	57 ± 11	0.003
Male	724 (47.4)	418 (46.5)	188 (46.4)	118 (52.7)	0.232
Duration of diabetes (years)	9.2 ± 7.7	7.1 ± 6.6	12.2 ± 8.3	11.9 ± 7.9	<0.001
BMI (kg/m^2^)	24.9 ± 3.7	25.3 ± 3.6	24.6 ± 3.7	23.5 ± 3.7	<0.001
SBP (mmHg)	130.5 ± 19.4	129.0 ± 18.5	132.0 ± 20.3	133.6 ± 21.2	0.014
DBP (mmHg)	77.7 ± 13.1	76.9 ± 12.8	78.5 ± 12.9	79.6 ± 14.2	0.063
FPG (mg/dl)	151.4 ± 72.8	143.9 ± 64.4	157.3 ± 71.8	170.9 ± 97.7	<0.001
HbA1c (%)	7.8 ± 1.8	7.4 ± 1.7	8.3 ± 1.9	8.3 ± 2.1	<0.001
Hemoglobin (g/dl)	13.1 ± 1.8	13.5 ± 1.6	12.7 ± 1.9	12.0 ± 1.9	<0.001
Total cholesterol (mg/dl)	167.2 ± 39.0	167.2 ± 35.0	166.4 ± 44.1	168.5 ± 44.0	0.826

Data are expressed as mean ± standard deviation and number (percent). Abbreviation: DR: diabetic retinopathy; NPDR: nonproliferative diabetic retinopathy; PDR: proliferative diabetic retinopathy; BMI: body mass index; SBP: systolic blood pressure; DBP: diastolic blood pressure; FPG: fasting plasma glucose; Hemoglobin A1c: HbA1c.

**Table 2:** Comparison of characteristics by progression of diabetic retinopathy (*n* = 1303).

Characteristics	Non-progression, *N* = 1169	Progression, *N* = 134	*p* value
Age (years)	59 ± 11	57 ± 12	0.060
Male	545 (46.6)	61 (45.5)	0.809
Duration of diabetes (years)	8.5 ± 7.5	10.6 ± 7.8	0.002
BMI (kg/m^2^)	25.1 ± 3.6	24.9 ± 3.7	0.560
SBP (mmHg)	130.1 ± 19.2	128.5 ± 18.0	0.467
DBP (mmHg)	77.6 ± 12.8	76.4 ± 12.1	0.433
FPG (mg/dl)	145.2 ± 64.1	173.6 ± 83.8	<0.001
HbA1c (%)	7.6 ± 1.7	8.7 ± 2.0	<0.001
eGFR (ml/min/1.73m^2^)	78.2 ± 21.6	77.0 ± 22.9	0.520
eGFR category			
G1 (≥90 ml/min/1.73m^2^)	303 (25.9)	34 (25.4)	0.483
G2 (60-89 ml/min/1.73m^2^)	664 (56.8)	70 (52.2)	
G3 (30-59 ml/min/1.73m^2^)	176 (15.1)	27 (20.1)	
G4 (15-29 ml/min/1.73m^2^)	26 (2.2)	3 (2.2)	
Albuminuria^∗^			
-	979 (91.8)	95 (81.2)	<0.001
+~+++	88 (8.2)	22 (18.8)	
Hemoglobin (g/dl)	13.3 ± 1.7	13.2 ± 1.8	0.782
Total cholesterol (mg/dl)	167.5 ± 37.5	165.2 ± 43.4	0.581

Data are expressed as mean ± standard deviation and number (percent). ^∗^1184 subjects measured albuminuria on urine dipstick. Abbreviation: DR: diabetic retinopathy; NPDR: nonproliferative diabetic retinopathy; PDR: proliferative diabetic retinopathy; BMI: body mass index; SBP: systolic blood pressure; DBP: diastolic blood pressure; FPG: fasting plasma glucose; Hemoglobin A1c: HbA1c; eGFR: estimated glomerular filtration rate.

**Table 3:** Logistic models for DR progression.

Characteristics	From no DR to NPDR		From NPDR to PDR	
	Univariate	Multivariate	Univariate	Multivariate
	Odds ratio (95% CI)	Odds ratio (95% CI)	Odds ratio (95% CI)	Odds ratio (95% CI)
Age (per year)	0.999 (0.981-1.018)		0.953 (0.926-0.980)^∗^	0.966 (0.937-0.995)^∗^
Duration of diabetes (per year)	1.067 (1.037-1.098)^∗^	1.062 (1.025-1.100)^∗^	0.995(0.995-1.037)	
FPG (per mg/dl)	1.006 (1.003-1.008)^∗^	1.003 (1.000-1.007)	1.003 (0.999-1.007)	
HbA1c (per %)	1.401 (1.261-1.557)^∗^	1.353 (1.191-1.537)^∗^	1.210 (1.028-1.426)^∗^	1.111 (0.933-1.323)
Albuminuria (vs. no)	3.843 (1.889-7.819)^∗^	2.791 (1.244-6.263)^∗^	1.993 (0.908-4.376)	
eGFR (per ml/min/1.73m^2^)	0.993 (0.982-1.003)		0.998 (0.983-1.013)	
Decrease eGFR > 20% (vs. no)	1.953 (1.005-3.796)^∗^	0.879 (0.373-2.075)	3.423 (1.696-6.908)^∗^	2.553 (1.219-5.348)^∗^

^∗^ *p* < 0.05. Abbreviation: DR: diabetic retinopathy; NPDR: nonproliferative diabetic retinopathy; PDR: proliferative diabetic retinopathy; FPG: fasting plasma glucose; hemoglobin A1c: HbA1c; eGFR: estimated glomerular filtration rate.

## Data Availability

The authors confirm that the data supporting the findings of this study are available within its supplementary materials.
